# Special Issue of Nanomaterials: Mechanics of Nanostructures and Nanomaterials

**DOI:** 10.3390/nano12030476

**Published:** 2022-01-29

**Authors:** Krzysztof Kamil Żur, Ali Farajpour

**Affiliations:** 1 Faculty of Mechanical Engineering, Bialystok University of Technology, 15-351 Bialystok, Poland; 2 School of Mechanical Engineering, University of Adelaide, Adelaide 5005, Australia; ali.farajpourouderji@adelaide.edu.au

Nanostructures have shown great potential to be used as the building components of many nanoelectromechanical and microelectromechanical systems. In a remarkable number of these ultrasmall devices, understanding the mechanical characteristics is crucial in order to improve the efficiency of the target system. Overall, there are three main approaches for obtaining the mechanical characteristics at nanoscales: (1) experimental techniques, (2) molecular dynamics (MD) simulations, and (3) size-dependent continuum modelling. The second and third approaches are commonly utilised as theoretical tools to explain the underlying reasons behind experimentally observed patterns and to extract the mechanical characteristics where performing reliable experiments is difficult. 

In this Special Issue, recently developed experimental techniques, MD simulations, and size-dependent continuum models of structural elements at nanoscales are discussed. Nanoscale structural elements include but are not limited to carbon nanotubes, sliver nanobeams, piezoelectric nanowires, boron nitride nanotubes, and graphene sheets. Possible directions for future works on the mechanical behaviour of structures at nanoscales based on different nonlocal elasticity theories are highlighted. In particular, original articles about the mechanical properties of nanostructures and their responses to multifield loads were welcomed.

In the presented Special Issue, six research papers [1,2,3,4,5,6] are published. Topics of published papers cover analyses of different engineering problems of nanostructures. The range of themes addressed in this Special Issue is certainly not exhaustive. The scope of applications of nanostructured materials and structures in diverse environments has been broadening rapidly. Many more complex experimental, theoretical, and numerical investigations are still needed. We hope that this Special Issue will provide the reader with a state-of-the-art perspective on some current research thrusts that use different techniques to analyse and control properties of nanoscale structures.
